# Comparison between Cystatin C- and Creatinine-Based Estimated Glomerular Filtration Rate in the Follow-Up of Patients Recovering from a Stage-3 AKI in ICU

**DOI:** 10.3390/jcm11247264

**Published:** 2022-12-07

**Authors:** Fateme Nateghi Haredasht, Liesbeth Viaene, Celine Vens, Nico Callewaert, Wouter De Corte, Hans Pottel

**Affiliations:** 1 Department of Public Health and Primary Care, KU Leuven, Campus KULAK, Etienne Sabbelaan 53, 8500 Kortrijk, Belgium; 2 ITEC-Imec, KU Leuven, Etienne Sabbelaan 51, 8500 Kortrijk, Belgium; 3 Department of Nephrology, AZ Groeninge Hospital, President Kennedylaan 4, 8500 Kortrijk, Belgium; 4 Laboratory Department, AZ Groeninge Hospital, President Kennedylaan 4, 8500 Kortrijk, Belgium; 5 Department of Anesthesiology and Intensive Care Medicine, AZ Groeninge Hospital, President Kennedylaan 4, 8500 Kortrijk, Belgium

**Keywords:** acute kidney injury, intensive care unit, serum creatinine, serum cystatin C, eGFR, chronic kidney disease

## Abstract

Background: Acute kidney injury (AKI) in critically ill patients is associated with a significant increase in mortality as well as long-term renal dysfunction and chronic kidney disease (CKD). Serum creatinine (SCr), the most widely used biomarker to evaluate kidney function, does not always accurately predict the glomerular filtration rate (GFR), since it is affected by some non-GFR determinants such as muscle mass and recent meat ingestion. Researchers and clinicians have gained interest in cystatin C (CysC), another biomarker of kidney function. The study objective was to compare GFR estimation using SCr and CysC in detecting CKD over a 1-year follow-up after an AKI stage-3 event in the ICU, as well as to analyze the association between eGFR (using SCr and CysC) and mortality after the AKI event. Method: This prospective observational study used the medical records of ICU patients diagnosed with AKI stage 3. SCr and CysC were measured twice during the ICU stay and four times following diagnosis of AKI. The eGFR was calculated using the EKFC equation for SCr and FAS equation for CysC in order to check the prevalence of CKD (defined as eGFR < 60 mL/min/1.73 m^2^). Results: The study enrolled 101 patients, 36.6% of whom were female, with a median age of 74 years (30–92), and a median length of stay of 14.5 days in intensive care. A significant difference was observed in the estimation of GFR when comparing formulas based on SCrand CysC, resulting in large differences in the prediction of CKD. Three months after the AKI event, eGFR_CysC_ < 25 mL/min/1.73 m^2^ was a predictive factor of mortality later on; however, this was not the case for eGFR_SCr_. Conclusion: The incidence of CKD was highly discrepant with eGFR_CysC_ versus eGFR_SCr_ during the follow-up period. CysC detects more CKD events compared to SCr in the follow-up phase and eGFR_CysC_ is a predictor for mortality in follow-up but not eGFRSCr. Determining the proper marker to estimate GFR in the post-ICU period in AKI stage-3 populations needs further study to improve risk stratification.

## 1. Introduction

Acute kidney injury (AKI) is a common clinical syndrome characterized by a rapid decline in kidney function [1,2,3,4]. Two new classification definitions of AKI were proposed in 2004 and 2007, Risk, Injury, Failure, Loss of kidney function, and End-stage kidney disease (RIFLE) and Acute Kidney Injury Network (AKIN), respectively. In 2012, Kidney Disease Improving Global Outcomes (KDIGO) published a clinical guideline to harmonize AKIN and RIFLE diagnostic criteria into one common diagnostic guideline [5]. AKI, particularly AKI stage 3, is associated with a significant increase in mortality, as well as short-term and long-term renal dysfunction, which may ultimately lead to chronic kidney disease (CKD). The prediction of these events (AKI and CKD after AKI) has gained greater attention in recent years. The basis for a good prediction model for AKI and/or CKD is built upon the predictability and the range of values of biomarkers that are taken into consideration by the model.

Currently, serum creatinine (SCr) is the most widely used biomarker to estimate glomerular filtration rate (GFR), which is the best overall index of kidney function [6]. Nevertheless, SCr has some limitations, since it depends on muscle mass. Consequently, SCr-based eGFR equations may overestimate the true GFR of critically ill patients, since these patients suffer from continuous loss of muscle mass.

In addition to SCr, cystatin C (CysC) is another biomarker of kidney function which has attracted the attention of researchers and clinicians in recent years. Even though CysC can also be affected by non-GFR determinants, the non-GFR determinants that affect CysC are distinct from those that affect SCr. Smoking status and serum C-reactive protein level are, for instance, independently associated with serum CysC levels [7]. While SCr-based eGFR-equations are widely used, several CysC-based eGFR-equations have been validated [8,9,10]. The most commonly used equations to calculate eGFR include the Chronic Kidney Disease Epidemiology Collaboration (CKD-EPI) equation [11], and the full age spectrum (FAS) equation [9,12]. Recently, Pottel et al. introduced an optimized FAS SCr-based equation, named the European Kidney Function Consortium (EKFC) equation, to estimate GFR [13].

Predicting CKD following AKI is a highly important yet missing topic in the AKI research field. The earlier CKD is diagnosed after AKI, the less intensive utilization of resources and the better the prevention of morbidity and mortality [14]. Delanaye et.al. [15] compared the performance of CysC and SCr as biomarkers for estimating GFR in 47 critically ill patients and concluded that CysC significantly outperformed SCr for the detection of an impaired GFR. Moreover, a recent study has been conducted on 22,488 critically ill patients to compare long-term mortality risk prediction by eGFR using an SCr-based equation (CKD-EPI), a CysC-based equation (CAPA) [10], and a composite SCr/CysC-based equation (CKD-EPI). Results showed that the single-biomarker CysC equation performed better compared to the SCr or composite equations when estimating GFR for risk prediction purposes in critically ill patients [16]. Moreover, Gharaibeh et al. claimed that CysC decreases before SCr in most hospitalized patients with acute kidney injury and therefore predicts renal recovery earlier than SCr [17]. Despite evidence confirming the superiority of CysC over SCr in detecting AKI in the intensive care unit (ICU), there is no similar evidence comparing CysC to SCr in detecting CKD in the post-ICU period after experiencing AKI in ICU. Researchers have mostly used SCr to estimate GFR in studies that attempted to detect renal recovery or CKD in survivors of AKI after discharge. To our knowledge, only one study by Rimes-Stigare et al. [18] focused on the occurrence of CKD and acute kidney disease (AKD) in AKI survivors three months after AKI that used both SCr and CysC. Their result showed significant renal impairment at least 25% according to SCr-based CKD and 67% when classified using CysC-estimated GFR.

In this study, we investigated whether the eGFR based on CysC differed from the eGFR based on SCr during the ICU stay and the follow-up period in adult ICU patients who had experienced an AKI stage-3 event. Additionally, we examined whether the eGFR determined by using the CysC-based FAS equation would further improve the correlation between eGFR and CKD diagnosis, as well as adjusted risks of death in the follow-up, compared to the use of an SCr-based eGFR. We hypothesized that CysC might provide additional benefits in post-ICU and be better-related to the outcomes (CKD and mortality), considering the inherent risk of muscle wasting that affects SCr.

## 2. Materials and Methods

### 2.1. Study Design and Participants

This study is a prospective observational study where we used medical records of ICU patients aged > 18 years who were diagnosed with AKI stage 3 during their ICU stay in AZ Groeninge Hospital in Kortrijk, Belgium, between September 2018, and October 2020.

Exclusion criteria were patients with a baseline eGFR < 30 mL/min/1.73 m^2^ estimated by CKD-EPI [11], patients with renal replacement therapy (RRT) initiated before admission to the ICU, patients with a kidney transplant, patients with therapy restrictions with shift to palliative care, and patients who received extracorporeal blood purification techniques for reasons other than AKI. Demographic data, comorbidity data, the severity of illness scores (APACHE 2), admission diagnosis, laboratory data, and data concerning kidney function (serial SCr measurements, oliguria, the time when AKI stage 3 developed, urinary analysis, etc.) were reported during the ICU stay. These data were augmented with regular GFR estimation by the CKD-EPI formula using both SCr and CysC biomarkers. SCr measurements in clinical practice in ICU were collected every morning; however, CysC measurements were not part of routine clinical practice and were obtained only at the time of admission to ICU and at the time of diagnosis of AKI (in most patients with a limited time lag). Furthermore, these patients have been followed-up at the nephrology department at 3, 6, 9, and 12 months after AKI stage-3 diagnosis in ICU. During these follow-up visits, the eGFR again was determined using both biomarkers.

### 2.2. Definitions—Acute Kidney Injury Criteria and Calculations

KDIGO criteria for AKI stage 3 have been used for the inclusion of patients based on SCr or urine output (UO). KDIGO defines stage 3 as an increase in SCr up to 3 times from baseline within a 7-day period or UO < 0.3 mL/kg/h for ≥24 h [5]. In this study, true baseline SCr was available for patients who had an SCr measurement from an earlier visit (previous to their hospital or ICU admission). In the absence of such records, baseline SCr was considered the first record of a patient’s hospitalization prior to being admitted to the ICU.

### 2.3. Serum Creatinine and Cystatin C Measurement

All SCr measurements were performed with an Enzymatic method that is traceable to the isotope dilution mass spectrometric method (IDMS), which is the internationally approved reference method for measuring creatinine. In addition, CysC concentrations were measured by Liège University Hospital using a particle-enhanced nephelometric immunoassay on the BNII nephelometer (Siemens Healthcare Diagnostics, Marburg, Germany). The assay was calibrated against the international certified reference material ERM-DA471/IFCC for CysC.

### 2.4. Evaluation of Glomerular Filtration Rate

The SCr-based estimated glomerular filtration rate (eGFR_SCr_) was calculated according to the EKFC equation introduced by Pottel et al. in 2021 [13]:

(1) $$\begin{array}{l} \mathrm{EKFC}-\mathrm{eGFR}\\ =\{\begin{array}{c} \;107.3\times {\left(\mathrm{SCr}/Q_{\mathrm{crea}}\right)}^{-0.322}\left[\times 0.990^{\left(\mathrm{Age}-40\right)}\;\mathrm{if}\;\mathrm{age}>40\;\mathrm{years}\right],\;\;\mathrm{SCr}/Q_{\mathrm{crea}}<1\\ \;107.3\times {\left(\mathrm{SCr}/Q_{\mathrm{crea}}\right)}^{-1.132}\;\left[\times 0.990^{\left(\mathrm{Age}-40\right)}\;\mathrm{if}\;\mathrm{age}>40\;\mathrm{years}\right],\;\mathrm{SCr}/Q_{\mathrm{crea}}\geq 1 \end{array} \end{array}$$

The EKFC equation is based on normalized SCr ($SCr/Q_{crea}$), where $Q_{crea}$ is the median SCr from healthy populations, which is 0.70 mg/dL for females and 0.90 mg/dL for males.

The CysC-based estimated glomerular filtration rate (eGFR_CysC_) was calculated according to the full age spectrum (FAS) equation introduced by Pottel et al. in 2017 [9,12]:

(2) FAS−eGFR={  107.3CysCQCys ; for 2≤ age ≤40 years 107.3CysCQCys×[0.988(Age−40)]; for age>40 years

The FAS equation is based on normalized CysC ($\mathrm{CysC}/Q_{Cys})$, where $Q_{Cys}$ is the median CysC from healthy populations, which is 0.82 mg/L when age <70 years and 0.95 otherwise, both for males and females.

### 2.5. Outcomes

The primary outcome was the post-ICU incidence of CKD after experiencing AKI stage 3, based on decreased eGFR detected by SCr and CysC levels.

To evaluate whether post-ICU eGFR values measured by each marker were clinically valid, we compared associations of eGFR, detected by CysC versus SCr level, with mortality after ICU discharge as a clinical endpoint.

### 2.6. Statistical Analyses

Continuous variables were presented as medians with interquartile ranges (IQR) and categorical variables were expressed as percentages. Correlation between all measurements of the biomarkers was assessed using Pearson and Spearman’s correlation coefficient. The normality of the distributions was assessed with the Shapiro-Wilk test. A Mann-Whitney U test/Wilcoxon rank-sum test was used to compare continuous variables of independent subgroups.

The associations of eGFR_SCr_ and eGFR_CysC_ with mortality were analyzed using Cox proportional hazard regression and logistic regression models, adjusted for covariates age, sex, length of stay (LoS), and dialysis in ICU.

Kaplan–Meier survival curves were plotted for SCr- or CysC-based eGFR < 25 and eGFR ≥ 25 mL/min/1.73 m^2^ in the first follow-up measurement and compared using the log-rank test.

Given the multiple visits per patient during follow-up, we used linear mixed models, derived slopes and intercepts for both eGFR_SCr_ and eGFR_CysC_ (only for the follow-up period), and compared the slopes and intercepts. For mixed-effect models, subjects and time (days in follow-up) were treated as random effects. Supplementary Materials provide more details about the statistical analysis. A two-tailed *p*-value of *p* < 0.05 was considered statistically significant. Analyses were carried out using R Statistical Software (version 4.0.5) [19].

## 3. Results

### 3.1. Patients

A total of 101 critically ill patients (37 females, median (IQR) age of 74 (30–92) years) who developed AKI stage 3 were included in this study. Characteristics of patients on ICU admission and after discharge are shown in Table 1. Patients who survived ICU and who were followed-up successfully with no dropout had six different measurements of both SCr and CysC: at the time of admission to ICU, at the time of developing AKI stage 3, and four follow-up times (every three months up to one year after AKI diagnosis). A 45% proportion of the cohort (n = 46) patients received dialysis, with a median of 13 (1–160) days during ICU stay, and the mortality rate during the study was 42.6% (n = 43). A total of 24 patients died during ICU stay and 19 patients died in the follow-up phase, of which 2 died between ICU discharge and the first follow-up. The number of patients attending follow-up visits decreased due to patient dropouts and mortality. Table 2 summarizes the median days after hospital discharge together with the number of SCr, CysC, and both SCr/CysC measurements in each follow-up visit. In follow-up visits, the number of patients with SCr and CysC measurements may not match due to storage and transport issues.

### 3.2. Correlation between Serum Creatinine and Cystatin C

**Figure 1:**
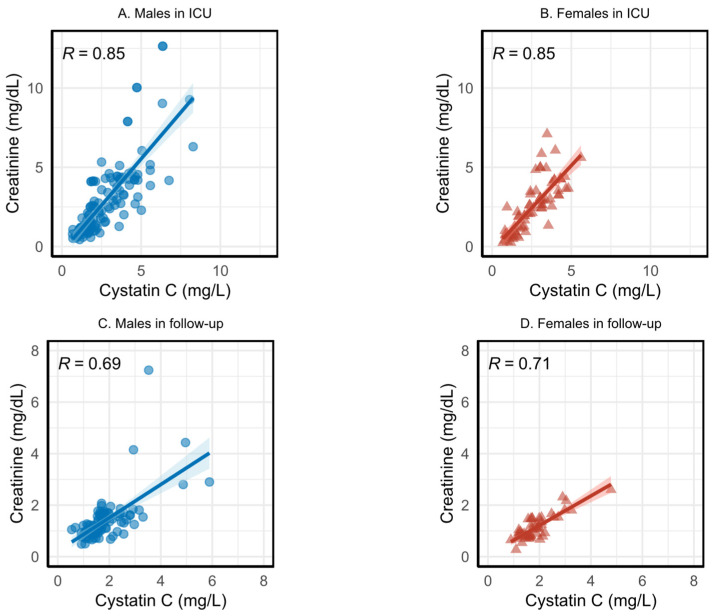
Spearman correlation coefficient for males and females for serum creatinine (SCr) and cystatin C (CysC) in the intensive care unit (ICU) stay and follow-up phases. (**A**,**B**) show correlation coefficient for males and females for SCr and CysC in the ICU stay, respectively. (**C**,**D**) shows correlation coefficient for males and females for SCr and CysC in the follow-up phase, respectively.

Figure 1 shows the Spearman correlation between SCr and CysC, before any adjustment (for age and/or sex), during ICU stay, and during the follow-up phase using all measurements. As shown in Figure 1, SCr and CysC are positively related, but CysC levels off at 8 mg/L while SCr can rise higher than 15 mg/dL. Since SCr and CysC tend to move in the same direction, but not necessarily at the same rate, their relationships are monotonic. Due to the monotonic relationship between the variables, we chose the Spearman correlation coefficient in Figure 1.

In Figure 1, graphs A and B show the relation between SCr and CysC during ICU stay for males and females, respectively, and graphs C and D show the relation during the follow-up phase for males and females, respectively. Figure 1 illustrates the relatively high correlation between the two biomarkers during ICU; however, the correlation during the follow-up phase is much lower, especially for males. Note that the axis values are different during ICU and the follow-up. Rescaling SCr to SCr/Q and CysC to CysC/Q did not change the correlation coefficients.

### 3.3. Evaluation of eGFR Using Serum Creatinine and Cystatin C

**Figure 2:**
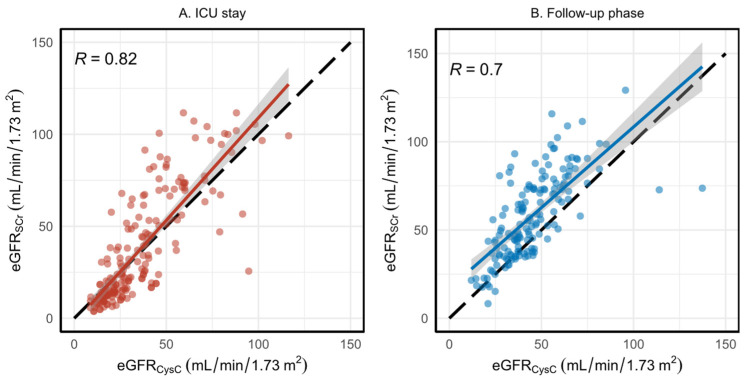
Estimated glomerular filtration rate (eGFR) comparison using SCr and CysC in ICU stay and follow-up phase. (**A**) shows the comparison during ICU stay and (**B**) sows the comparison during follow-up phase. The red and blue curves are fitted linear regression models in ICU and follow-up, respectively, and the faded zones are the confidence intervals around the lines. The black dashed line shows the identity line.

Results of eGFR_SCr_ against eGFR_CysC_ during ICU stay and follow-up period are presented in Figure 2A,B. The Pearson’s correlation coefficient between the two eGFRs during the ICU stay (plot A) is 0.82; however, during the follow-up phase (plot B) the correlation decreased to 0.7. We used the Pearson’s test since both equations are supposed to predict the same value (GFR); hence, they should be linearly related, ideally with a correlation coefficient of ‘1’ and slope = 1 and intercept = 0. However, our results demonstrate systematic deviation from the identity line, showing that the two biomarkers behave significantly differently in the follow-up phase (*p*-value < 0.0001; Wilcoxon test). No significant deviation from the identity line was observed during the ICU stay (*p*-value = 0.1; Wilcoxon test). Furthermore, the Bland–Altman analysis for the ICU stay and follow-up phase is presented in Supplementary Material (Figure S1). We see that, during ICU stay, the average difference between eGFR_SCr_ and eGFR_CysC_ is near-zero; however, it is nearly 15 in the follow-up phase.

**Figure 3:**
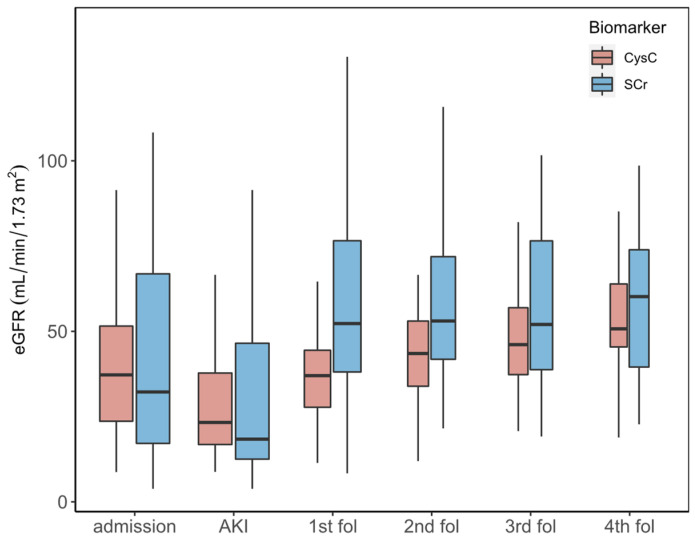
Comparison of estimated glomerular filtration rate using serum creatinine (eGFR_SCr_) and estimated glomerular filtration rate using cystatin C(eGFR_CysC_) during ICU stay and each follow-up phase.

Figure 3 shows boxplots for all measurements of eGFR based on SCr and CysC during ICU stay (at admission and AKI event time) and during each follow-up visit. Results show that eGFR_SCr_ levels are higher during each follow-up visit compared to eGFR_CysC_. Notably, the number of patients differs in each follow-up visit due to drop-out or death.

Table S1 in the Supplementary Material provides data for the median eGFR value and interquartile range (IQR) for patients with both biomarkers measured. There is no statistically significant difference between the two eGFR values during ICU stay (Wilcoxon signed-rank test); however, the difference is significant during each follow-up visit (Wilcoxon signed-rank test) except for the last follow-up (fourth), which is probably due to the small sample size.”

**Figure 4:**
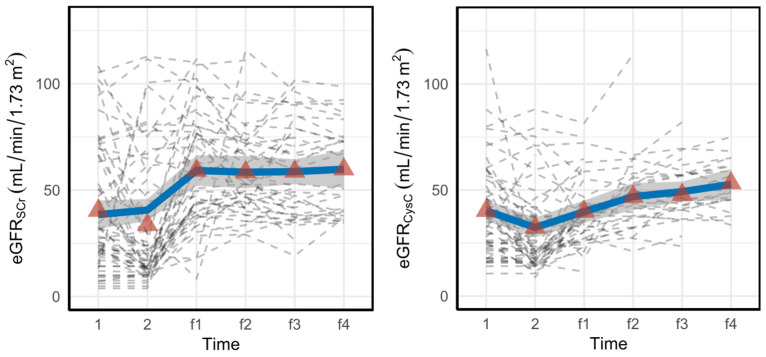
Within-subject evolution of eGFR for alive patients from the first day in ICU until the last follow-up. The dashed gray lines represent each subject, the red triangles show the average eGFR values at that specific time point, and the blue lines are smooth curves obtained via locally estimated scatterplot smoothing (LOESS). The gray band is a 95% confidence band for the regression line.

The within-subject evolution of the eGFR for alive patients using both SCr and CysC from the first measurements in ICU until the last follow-up is shown in Figure 4. We used the measurements of only alive patients since only the survivors could have data at the latest visits (some curves end before the follow-up moment due to dropouts).

As shown in Figure 4, eGFR_CysC_ increases steadily from the time AKI is diagnosed (time point 2) onward, whereas eGFR_SCr_ plateaus at the first follow-up (time point f1).

Figure S1 in the Supplementary Material shows the individual weight evolution during the follow-up period. Results show that the patients start gaining weight after the second follow-up.

Mixed-model analysis (Table 3) during follow-up was performed: eGFRSCr starts at a much higher average level but shows no change over time during the follow-up period, while eGFRCysC is lower at month 3 (first FU), but shows a significant increase during follow-up. Moreover, the intercepts are significantly different since the 95% confidence intervals (CI) do not overlap. Table 3 supports our hypothesis regarding the effect of muscle mass on SCr Considering that the intercept for eGFR_SCr_ in the mixed-effect model is much higher than the intercept for eGFR_CysC_ and furthermore that the slope for eGFR_SCr_ is not changing while the slope for eGFR_CysC_ is increasing (regaining kidney function) confirms that according to the SCr, it seems as if the kidneys have already recovered at the first follow-up, while CysC-based eGFR still shows ongoing recovery during follow-up.

Table 4 indicates the number of patients with eGFR < 60 mL/min/1.73 m^2^ and eGFR ≥ 60 mL/min/1.73 m^2^ based on SCr and CysC in each follow-up visit. We see large differences in the incidence of chronic kidney disease (defined as eGFR < 60 mL/min/1.73 m^2^) using the two biomarkers during follow-up visits. Specifically, in the first follow-up, as shown in Table S2 in Supplementary Material, 19 patients were classified as having CKD using eGFR_CysC_; however, eGFR_SCr_ had classified them as having no CKD. The difference between the two biomarkers in detecting CKD in the first follow-up was statistically significant (McNemar’s chi-squared = 14.45, p−value=0.000143).

### 3.4. The Associations between eGFR and Outcome

We also evaluated whether there were differences between those with CKD and those without CKD based on either eGFR SCr or CysC levels during each follow-up time (Tables S3–S6 in Supplementary Material). Stages of CKD are defined using the KDIGO guidelines. The results of each follow-up show a large difference in the patients’ classification of CKD using SCr and CysC. For instance, according to Table S3 in Supplementary Material, eGFR_SCr_ classifies the majority of patients (n = 19) as GFR category 2, on the other hand for eGFR_CysC_, the majority (n = 29) belong to class CKD3B (moderate to severely decreased).

There were 43 (42.6%) deaths during the study, of which 24 occurred during the ICU stay and 19 during the follow-up period. Univariate Cox proportional hazard regression models were performed to examine the risk factors associated with mortality in ICU with all patients included and mortality in follow-up with patients who survived ICU (Table 5).

The analysis of the association between eGFR and mortality in ICU was performed on the whole population (n = 101), and we considered variables of age, gender, average eGFR_CysC_ in ICU, and average eGFR_SCr_ in ICU. The average eGFR_CysC/SCr_ in ICU is the average over two eGFRs using SCr and CysC during ICU stay.

In analyses of the association between eGFR and mortality in the follow-up phase, patients who survived the ICU and appeared at the first follow-up visit were included. Variables including age, gender, length of stay in ICU (LoS ICU), dialysis in ICU, and reduced eGFR_CysC_ and eGFR_SCr_ in the first follow-up were considered in the model. Reduced eGFR_CysC/SCr_ in the first follow-up was defined as eGFR < 25 mL/min/1.73 m^2^.

**Figure 5:**
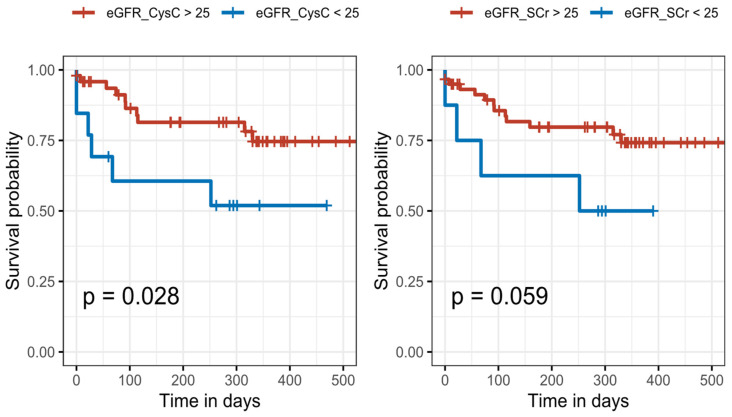
Kaplan–Meier survival curves according to eGFR levels using CysC (**left**) and SCr (**right**) in patients with eGFR below and above 25 mL/min/1.73 m^2^ in the first follow-up measurement. There were 49 deceased patients with eGFR_CysC_ below 25 and 13 with eGFR_CysC_ above 25, and 60 deceased patients with eGFR_SCr_ below 25 and 8 with eGFR_SCr_ above 25.

In Table 5, significant risk factors are denoted in bold and with a “*” that represents the significance code as p < 0.05. Results of Cox proportional hazard regression models in Table 5 demonstrate that age is a significant risk factor for mortality in ICU and that a patient who has eGFR based on CysC below 25 mL/min/1.73 m^2^ at first follow-up, has a significantly increased risk for mortality compared to a patient who has eGFR based on SCr below 25 mL/min/1.73 m^2^. Kaplan–Meier curves using eGFR <25 and ≥25 as strata illustrate these findings (Figure 5). We divided the patients who survived during ICU into two groups based on eGFR value at first follow-up using both biomarkers to investigate whether having an eGFR below or above 25 mL/min/1.73 m^2^ is predictive of mortality during follow-up (Figure 5).

## 4. Discussion

AKI is very common in the critically ill. Spontaneous resolution (or rapid response to treatment) occurs in some patients, even after experiencing the most severe AKI Stage-3 event. Despite its relative non-specificity, SCr remains the gold-standard for defining AKI and for follow-up after an AKI event. However, less is known about CysC during the follow-up phase after experiencing an AKI episode. The present study investigates whether the use of CysC has advantages over SCr as a biomarker for renal function for adult ICU patients who had experienced such an AKI stage-3 event. First, by comparing the evolution of the two biomarkers and estimated GFR during the ICU stay and post-AKI follow-up, we discovered that they behave differently after the ICU discharge, and the correlation between the two GFR estimates drops during the follow-up period. Several articles suggest that SCr may result in an overestimation of recovery by ignoring the decrease in SCr due to the loss of muscle mass that occurs during critical illness [20,21]. The majority of our AKI stage-3 patients (almost 30%) developed this event on the day of admission to the ICU or the day following admission; therefore, SCr was not affected by the loss of muscle mass during the ICU stay, resulting in a higher correlation between SCr-based and CysC-based eGFR during the stay.

Using different statistical analyses, we compared creatinine- and cystatin C-based estimates of GFR during ICU stay and the follow-up period. On admission, we observed that both eGFRs are approximately the same, which strongly supports the hypothesis that the loss of muscle mass explains the differences observed over time in the follow-up phase. In the follow-up period, we found a significant difference in eGFR values between the two biomarkers. In particular, we saw that eGFR_CysC_ increases steadily from the time the AKI is diagnosed onward, whereas eGFR_SCr_ plateaus at the first follow-up, which may reflect the fact that kidney function improves (thus decreasing SCr) and muscle mass increases when patients are recovering from their ICU stay (thereby increasing SCr). The combined effect may be that SCr does not change much. Due to the fact that CysC is not affected by muscle mass, there is no double effect present for CysC.

Moreover, the occurrence of low eGFR_CysC_ in the follow-up after AKI was more frequent than the occurrence of low eGFR_SCr_, especially during the first visit, about 1 month after hospital discharge. Two phenomena might explain the high eGFR_SCr_ values in the first follow-up. First, patients may not yet have recovered from ICU stay, leading to a lower SCr (higher eGFR_SCr_). Second, patients recovering from AKI have their kidney function improving, which consequently leads to a lower SCr value (higher eGFR_SCr_). It was suggested in [20] that, although follow-up care pathways should be tailored to individual conditions, and reassessment of renal function 90 days after discharge from the hospital is more reasonable in order to allow time for the recovery of muscle mass as well as any further improvement of renal function. Although there was no significant increase from the first follow-up, our results also confirm that patients start gaining weight after the second follow-up. In addition to the effect of diet and muscle mass on creatinine production, overestimation of kidney function in AKI patients due to the elimination of creatinine by tubular secretion could explain these differences in eGFR_SCr_ and eGFR_CysC_ [22,23].

Furthermore, according to our results, CKD incidence was far higher when GFR was estimated using CysC than with SCr, which was a confirmation of the findings in the study by Rimes-Stigare et al. [18]. We observed that eGFR_SCr_ tends to classify more patients towards the less-severe stages compared to eGFR_CysC_. Both biomarkers give similar GFR estimates in the steady state, providing an acceptable correlation with measured GFR [24,25]. It is worth mentioning that we only considered the measurements in which both markers were measured. Our results suggest that patients may be classified differently according to the biomarker used. Differences in the incidence of CKD by the two biomarkers seen in our study could be related to a number of factors, including the loss of skeletal muscle mass and strength that occurs during an ICU stay and affects SCr levels even after discharge. It might also be due to the different abilities of CysC-based equations and SCr-based equations to estimate measured GFR in different populations such as elderly patients. Study results on the elderly have shown that, when SCr and CysC are combined, GFR estimates are more accurate and precise [25], while SCr-based equations are more inaccurate [26].

Since the surveillance of all patients would be expensive and impractical, we must establish how best to determine renal function during post-AKI follow-up. As in our results, we saw that a patient who has eGFR based on CysC below 25 mL/min/1.73 m^2^ at first follow-up has a significantly increased risk for mortality compared to a patient who has eGFR based on SCr below 25 mL/min/1.73 m^2^; hence, clinicians should look at eGFR_CysC_ instead of eGFR_SCr_ at the first follow-up. After evaluating different cut-offs, 25 was chosen because it gave us the best ‘survival’ discrimination between the two eGFR equations. Other cut-offs failed to reach significance probably because there were few participants. Future studies should validate this cut-off.

Even though our findings are intriguing and may be clinically useful, there are some limitations to be considered. First of all, the number of patients in our study was limited, and loss of follow-up is present and was due in part to logistical difficulties. Additionally, we did not measure GFR using a gold-standard technique since this is not routinely available and is practically impossible in an ICU setting. Moreover, our study was conducted in Europe, and our patient population consisted only of Caucasian patients. Thus, our results cannot be generalized to countries with predominantly black, Asian, or mixed-race populations; moreover, differences in SCr should be taken into consideration due to racial factors.

Furthermore, CysC is affected by inflammation and infection; however, we did not adjust CysC for this as CRP was not available in follow-up.

## 5. Conclusions

Our prospective observational study demonstrated that the incidence of CKD defined with CysC-based eGFR versus SCr-based eGFR during the follow-up period of critically ill patients, recovering from AKI stage 3 in their intensive care unit stay, was highly discrepant. In the follow-up phase, CysC-based eGFR categorized significantly more patients in more severe CKD stages than SCr-based eGFR, and eGFR_CysC_ was a better predictor of mortality compared to eGFR_SCr_. Accordingly, our study demonstrated that using SCr alone at follow-up could lead to an underestimate of renal dysfunction (CKD) among AKI stage-3 survivors. Further follow-up is required to evaluate the validity of estimated GFR based on both biomarkers by comparing them to clinical assessment and progression to dialysis.

## Figures and Tables

**Table 1:** Patient characteristics.

Characteristics	All Patients (N = 101)
Demographics	
Female sex, n (%)	37 (36.6%)
Age, years	74 (30−92)
Body weight, kg	83 (45−150)
Body mass index, kg/m^2^	27.7 (17–57)
ICU types	
MICU, n (%)	81 (80%)
SICU, n (%)	17 (16.8%)
Trauma, n (%)	3 (2.9%)
Admission diagnosis (%)	
Community-acquired pneumonia (CAP)	10%
Cardiac disease	9%
Acute respiratory failure	8%
Sepsis	7%
Aspiration pneumonia	6%
Pulmonary edema	4%
Other diagnosis	56%
Results	
Length of stay in ICU, days	14.5 (1–160)
Cystatin C (mg/L) at ICU admission	1.94 (0.67–8.06)
Creatinine (mg/dL) at ICU admission	1.98 (0.31–12.64)

ICU: intensive care unit; MICU: medical intensive care unit; SICU: surgical intensive care unit.

**Table 2:** Patients’ follow-up information after hospital discharge.

	First Follow-Up	Second Follow-Up	Third Follow-Up	Fourth Follow-Up
Number of survivors for follow-up	77 − 2 = 75	61	48	40
Number of dropouts	7	7	3	5
Median follow-up days	37	142	229	337
Number of patients with SCr values	68	54	45	35
Number of patients with CysC values	62	39	34	26
Number of patients with SCr and CysC	60	39	34	25

SCr: serum creatinine; CysC: serum cystatin.

**Table 3:** The output of the mixed-effect model results.

	eGFR_SCr_			eGFR_CysC_		
Predictors	Estimates	CI (95%)	p	Estimates	CI (95%)	p
Intercept	57.25	50.50–64.00	<0.001	37.85	33.8–42.27	<0.001
Slope	0.003	−0.01–0.02	0.7	0.041	0.01–0.07	0.004

CI: confidence interval.

**Table 4:** Number of patients with eGFR < 60 mL/min/1.73 m^2^ and eGFR ≥ 60 mL/min/1.73 m^2^ based on SCr and CysC in each follow-up visit.

	eGFR_SCr_		eGFR_CysC_	
	<60	≥60	<60	≥60
Visit 1 (n = 60)	34	26	52	8
Visit 2 (n = 39)	23	16	34	5
Visit 3 (n = 34)	20	14	29	5
Visit 4 (n = 25)	10	15	15	10

**Table 5:** Univariate Cox regression models for mortality in ICU and follow-up.

	Mortality in ICU		Mortality in Follow-Up	
Variable	HR	95% CI	HR	95% CI
Age	1.05 *	**1.01–1.09**	1.03	0.99–1.07
Gender (male)	0.93	0.41–2.13	2.022	0.67–6.10
LoS in ICU	-	-	0.98	0.96–1.01
Dialysis in ICU	-	-	1.86	0.87–3.95
Reduced eGFR_CysC_ in the first follow-up	-	-	3.32 *	**1.2–9.2**
Reduced eGFR_SCr_ in the first follow-up	-	-	2.8	0.91–8.65
Average eGFR_CysC_ in ICU	1	0.97–1.01	1.00	0.97–1.02
Average eGFR_SCr_ in ICU	0.98	0.96–1.01	1.00	0.98–1.02

Significant risk factors are denoted in bold and with a “*” that represents the significance code as *p* < 0.05.

## Data Availability

The data supporting the results of this study cannot be made publicly available due to the lack of approval from our ethics committee in this regard.

## Supplementary Materials

The following supporting information can be downloaded at: https://www.mdpi.com/article/10.3390/jcm11247264/s1, Figure S1. Individual trajectories for weight during the follow-up. The dashed gray lines represent each subject, the red triangles show the average weight values at that specific time point, and the blue lines are smooth curves obtained via LOESS; Table S1. eGFR (mL/min/1.73 m^2^) statistics by using biomarkers in ICU and follow-up phase. Table S2. Number of patients with eGFR < 60 mL/min/1.73 m^2^ and eGFR ≥ 60 mL/min/1.73 m^2^ based on SCr and CysC in the 1st follow-up visit. Table S3. Overview of patients with CKD stages according to eGFR using SCr and CysC during the 1st follow-up. Table S4. Overview of patients with CKD stages according to eGFR using SCr and CysC during the 2nd follow-up. Table S5. Overview of patients with CKD stages according to eGFR using SCr and CysC during the 3rd follow-up. Table S6. Overview of patients with CKD stages according to eGFR using SCr and CysC during the 4th follow-up.
