# Supplementary material for: Comparison between Cystatin C- and Creatinine-Based Estimated Glomerular Filtration Rate in the Follow-Up of Patients Recovering from a Stage-3 AKI in ICU
Source: J Clin Med. 2022 Dec 7;11(24):7264. doi: 10.3390/jcm11247264 (PMC9784749; doi:10.3390/jcm11247264)
Supplement: Supplementary file 1 [file jcm-11-07264-s001.zip › jcm-1978986-supplementary.pdf]

Comparison Between Cystatin C- and Creatinine-Based Estimated Glomerular Filtration Rate in the Follow-Up of Patients Recovering from a Stage-3 AKI in ICU

Supplementary Materials

Table S1. eGFR (mL/min/1.73m^2) statistics by using biomarkers in ICU and follow-up phase.

	ICU Stay				Follow-Up Phase							
	ICU Admission		AKI Diagnosis		1st Visit		2nd Visit		3rd Visit		4th Visit	
	Median	IQR	Median	IQR	Median	IQR	Median	IQR	Median	IQR	Median	IQR
CysC	37.2	27.9	23.3	21	37	16.3	43.5	19.1	45.7	19.6	51.8	19.2
SCr	32.2	49.7	18.5	34	50.2	39.4	55.8	27.9	52.5	40.2	67.4	34.1

ICU: intensive care unit; AKI: acute kidney injury; CysC: cystatin C; SCr; serum creatinine; IQR: interquartile range.

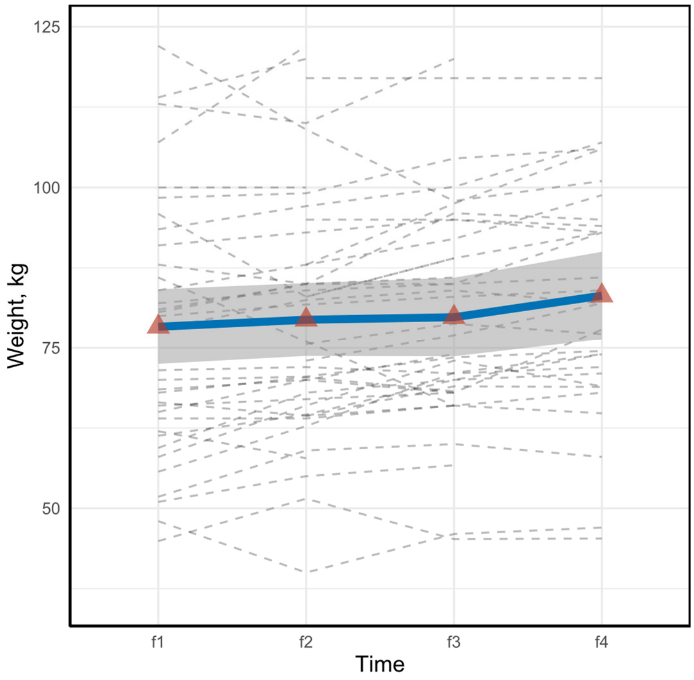

Figure S1. Individual trajectories for weight during the follow-up. The dashed gray lines represent each subject, the red triangles show the average weight values at that specific time point, and the blue lines are smooth curves obtained via LOESS.

Table S2. Number of patients with eGFR <60 mL/min/1.73m^2 and eGFR ≥60 mL/min/1.73m^2 based on SCr and CysC in the 1st follow-up visit.

**CKD**		eGFR$_{SCr}$		Total
		<60	≥60	
**eGFR$_{CysC}$**	**<60**	33	**19**	52
	≥60	1	7	8
Total		34	26	60

Table S3. Overview of patients with CKD stages according to eGFR using SCr and CysC during the 1st follow-up.

**CKD**		eGFR$_{SCr}$						
		CKD1	CKD2	CKD3A	CKD3B	CKD4	CKD5	Total
**eGFR$_{CysC}$**	CKD1	1	0	0	0	0	0	1
	CKD2	3	3	1	0	0	0	7
	CKD3A	2	4	1	0	0	0	7
	CKD3B	1	11	6	9	1	0	28
	CKD4	0	1	1	8	4	1	15
	CKD5	0	0	0	0	2	0	2
	Total	7	19	9	17	7	1	60

Table S4. Overview of patients with CKD stages according to eGFR using SCr and CysC during the 2nd follow-up.

**CKD**		eGFR$_{SCr}$						
		CKD1	CKD2	CKD3A	CKD3B	CKD4	CKD5	Total
**eGFR$_{CysC}$**	CKD1	0	1	0	0	0	0	1
	CKD2	1	3	0	0	0	0	4
	CKD3A	3	5	4	0	0	0	12
	CKD3B	0	3	7	5	0	0	15
	CKD4	0	0	3	2	1	0	7
	CKD5	0	0	0	0	1	0	1
	Total	4	12	14	7	2	0	39

Table S5. Overview of patients with CKD stages according to eGFR using SCr and CysC during the 3rd follow-up.

**CKD**		$eGFR_{SCr}$						
		CKD1	CKD2	CKD3A	CKD3B	CKD4	CKD5	Total
**$eGFR_{CysC}$**	CKD1	0	0	0	0	0	0	0
	CKD2	3	2	0	0	0	0	5
	CKD3A	1	7	3	2	0	0	13
	CKD3B	0	1	4	6	0	0	11
	CKD4	0	0	1	1	3	0	5
	CKD5	0	0	0	0	0	0	0
	Total	4	10	9	8	3	0	34

Table S6. Overview of patients with CKD stages according to eGFR using SCr and CysC during the 4th follow-up.

**CKD**		$eGFR_{SCr}$						
		CKD1	CKD2	CKD3A	CKD3B	CKD4	CKD5	Total
**$eGFR_{CysC}$**	CKD1	0	1	0	0	0	0	1
	CKD2	2	7	0	0	0	0	9
	CKD3A	0	4	2	3	0	0	9
	CKD3B	0	1	0	4	0	0	5
	CKD4	0	0	0	0	1	0	1
	CKD5	0	0	0	0	0	0	0
	Total	2	13	**2**	7	1	0	25
